# Pre-treatment functional connectivity patterns associated with variability in cognitive-behavioral therapy response in specific phobia

**DOI:** 10.1007/s00406-026-02313-y

**Published:** 2026-08-07

**Authors:** Judith Domínguez-Pérez, Judith Domínguez-Pérez, Francisco Rivero, Ascensión Fumero, Teresa Olivares, Rosario J. Marrero, Wenceslao Peñate

**Affiliations:** 1 Universidad de La Laguna, Santa Cruz de Tenerife, Spain; 2 Universidad Europea de Canarias, Santa Cruz de Tenerife, Spain

**Keywords:** fMRI, CBT, Specific phobia, Treatment-associated connectivity, Treatment response

## Abstract

Specific phobia is an anxiety disorder marked by excessive fear of particular objects or situations. Although cognitive-behavioral therapy is generally effective, patient response varies. The study aimed to explore neural predictors for characterization of treatment-response variability and individualized treatment approaches. Focusing on connectivity rather than structural or functional magnetic resonance imaging features, it also explored pre-treatment functional magnetic resonance imaging differences between high and low responders based on clinically significant changes after therapy. Twenty-nine adults with specific phobia to small animals underwent baseline functional magnetic resonance while exposed to real or virtual 3D images of phobic stimuli. Following an eight-week cognitive behavioral therapy, patients were classified as high or low responders based on clinically significant change. Pre-treatment functional connectivity was analyzed via region-of-interest-to-region-of-interest correlations. Two groups were formed (14 high responders and 15 low responders) and compared by a t-test. Low responders exhibited greater connectivity across language, cognitive-control, reward, and sensorimotor networks. Differences included hyperconnectivity between the nucleus accumbens, inferior frontal gyrus, superior temporal gyrus, and caudate, and between the medial prefrontal cortex and motor/sensorimotor regions. This profile suggests stronger integration of motivational, verbal-conceptual, and motor-preparatory systems, potentially reflecting heightened internal verbalization, rigid habit-based responses, and anticipatory motor readiness. The results therefore point toward a maladaptive pre-treatment connectivity state in low responders, marked by over-recruitment of self-referential and control networks that limit the capacity for flexible emotional updating during cognitive-behavioral therapy. These findings suggest that functional connectivity patterns may contribute to the identification of neural markers associated with treatment response.

## Introduction

Specific phobia is an anxiety disorder characterized by intense and disproportionate fear or anxiety toward specific objects or situations, lasting for several months and causing significant distress or impairment [1]. Cognitive processes associated with specific phobia include heightened threat anticipation, attentional bias toward feared stimuli, and maladaptive appraisal of danger [2–3].

Specific phobia has been successfully treated with different therapies such as gradual exposure, cognitive-behavioral therapy (CBT), acceptance and commitment therapy (ACT), or mindfulness [4–6]. Exposure can be delivered through multiple formats, including virtual exposure, which have demonstrated effectiveness comparable to traditional in-vivo exposure in several studies [7–8]. Despite the overall efficacy of CBT for specific phobia, not all individuals benefit equally from treatment. For some patients, CBT is not effective [9] and among those who do respond, the degree of benefit varies [10].

This variability in outcomes has led to increasing interest in identifying neural factors associated with therapeutic response. A recent systematic review and meta-analysis focusing on machine learning studies highlights that neuroimaging biomarkers are among the most promising predictors of CBT outcomes across various anxiety disorders [11]. This review, which included over 7,000 patients, found that models incorporating pre-treatment neuroimaging data reached a classification accuracy of approximately 74%, outperforming those based solely on clinical or demographic variables. These findings suggest that brain-based biomarkers, when analyzed using data-driven approaches such as machine learning, hold significant potential for guiding personalized interventions by identifying which patients are more likely to benefit from CBT.

Several studies have reported promising results using pre-treatment neuroimaging measures to predict CBT outcomes across anxiety disorders, with classification accuracies ranging from 79% to 92% [12–14].

However, subsequent studies have questioned the generalizability and clinical utility of these findings. Neuroimaging predictors have shown inconsistent performance across disorders, tasks, and analytic approaches, with some studies reporting low predictive accuracy or no added value beyond clinical and demographic variables [15–16]. These results challenge the idea that neural activity in emotional and cognitive-control networks reliably predicts treatment response, highlighting the need for further research on how brain markers interact with clinical features to predict CBT outcomes.

Likewise, clinical and demographic variables alone have shown limited predictive utility, suggesting that multimodal approaches incorporating neurobiological information may improve the characterization of treatment-response variability. These findings also highlight the complexity of treatment-related mechanisms and the need for larger multimodal datasets [9].

In summary, despite interest in neurobiological predictors, findings are mixed. Neuroimaging’s relationship with treatment response depends on the disorder, fMRI task, and validation in larger, multi-site datasets. Frequently, samples are divided into two groups: responders and non-responders to treatment. Among responders, there are different levels of clinical and statistical improvement, and these differences could also contribute to inconsistencies across findings, depending on criteria used to evaluate clinical significance [17]. Because structural and functional MRI findings have shown limited and inconsistent predictive utility, connectivity could be a relevant variable to consider. Functional connectivity between different brain regions could provide additional information about pre-existing differences between responders to psychological treatment and non-responders [18].

Importantly, the focus on functional connectivity is also grounded in previous neuroimaging work conducted by our group in specific phobia and CBT-related mechanisms. Prior studies using task-based fMRI paradigms have shown that phobic processing and CBT effects involve alterations in distributed functional networks including the insula, amygdala, cingulate cortex, precuneus, and prefrontal regions, suggesting that treatment-related neural changes may be better understood at the network level rather than solely in terms of isolated regional activation or structural alterations [19].

The present study was not designed to evaluate the efficacy of CBT itself, which is already well established in specific phobia, but rather to investigate pre-treatment functional connectivity differences associated with variability in treatment response.

The aim of this study was to identify neural characteristics associated with treatment response that could improve understanding of variability in treatment response. According to these findings and acknowledging that structural and functional MRI features showed a limited ability to predict treatment outcome, our study is focused on connectivity rather than structural or task-related MRI features. A secondary objective was to explore pre-treatment neural differences, as measured by fMRI, between patients who showed a high response to CBT (high responders) and those who showed a lower response (low responders), according to clinically significant changes.

## Methods and materials

### Participants

131 adults were recruited, of whom 78 were excluded due to health issues or missed sessions, 14 dropped out for reasons including non-attendance to MRI or therapy sessions, conditions incompatible with MRI procedures or withdrawal. 8 were excluded after fMRI realignment. The remaining 31 participants were classified as high or low responders based on several criteria regarding Situation-Response Inventory of Anxiousness (S-R inventory) pre- and post-treatment measures (see Data Analysis for the flow chart).

The inclusion criteria for participants were as follows:

- Being an adult with a specific phobia of spiders, cockroaches, or lizards.
- The specific phobia had to be the primary disorder and not attributable to another condition or phobia.
- Participants had not received any pharmacological and/or psychological treatment for their specific phobia in the last 12 months.
- Being right-handed.
- Having normal vision.
- Being medically able to undergo an fMRI session.

### Instruments

#### Clinical assessments

To verify phobia diagnoses, participants were administered the Composite International Diagnostic Interview (CIDI), Version 2.1 [20], using the Latin-American/Spanish cultural version [21]. Baseline phobia severity was assessed using the Situation-Response Inventory of Anxiousness (S-R inventory) [22]. This 14-item, 5-point Likert questionnaire records physiological, cognitive, and behavioral reactions to an anxiety-provoking stimulus. The inventory was completed at baseline, immediately after treatment, and again three months later. The inventory demonstrates excellent internal reliability (Cronbach’s α = 0.95). The S-R inventory was selected because it specifically assesses symptom dimensions directly associated with phobic anxiety and is sensitive to clinically meaningful changes following CBT interventions.

Anxiety severity in the presence of the feared animal was further evaluated with the Hamilton Anxiety Rating Scale (HAM-A) [23–24]. The scale demonstrates strong inter-rater reliability (Interclass Correlation Coefficient (ICC) = 0.74–0.96). A score ≥ 14 signified clinically significant anxiety. HAM-A was administered at baseline and post-treatment. It has high internal reliability (Cronbach’s α = 0.89).

Manual dominance was assessed using the Edinburgh Handedness Inventory [25]. According to Dragovic, its internal reliability is high (Cronbach’s α = 0.88) [26].

#### fMRI data acquisition

Functional and structural scans were obtained on a GE Signa Excite HD 3.0 T system (GE Healthcare, Madrid, Spain).

Functional series were collected with a Gradient-Echo EPI sequence: repetition time (TR) = 2000 ms, echo time (TE) = 30 ms, flip angle = 75°, field of view (FOV) = 25.6 cm, matrix = 64 × 64, 32 axial slices, voxel size = 4 × 4 × 4 mm.

Structural images used a 3-D T1-weighted FSPGR protocol: TR = 8852 ms, TE = 1756 ms, flip angle = 10°, FOV = 25.6 cm, matrix = 256 × 256, 172 slices, isotropic voxels of 1 mm (slice thickness = 1 mm, no inter-slice gap). Parallel imaging (ASSET) was applied, based on a calibration scan of 35 oblique (50°) slices acquired with TE = 2.1 ms, TR = 150 ms, matrix = 32 × 32, and 6 mm slice thickness.

A block design was employed, with each scanning run lasting approximately 11 min. Visual stimuli were delivered in stereoscopic format via MRI-compatible VisualStim™ digital 3-D goggles connected to a GeForce 8600 GT graphics card, ensuring paramagnetic isolation. Participants viewed 3-D video clips, either filmed or virtually rendered, showing spiders, cockroaches, and lizards in motion. All clips were presented against a uniform white background.

Functional neuroimaging images were processed and analyzed using SPM12 [27], a MATLAB-based tool widely used for fMRI and other neuroimaging data. It supports preprocessing-motion correction, spatial normalization, and smoothing and voxel-wise statistical modelling to detect activity differences across conditions or groups.

Participants with head motion exceeding 2 mm of translation or 2° of rotation were excluded from the analyses. Residual motion parameters obtained during realignment were included as nuisance regressors in first-level analyses.

### Procedure

The present study represents a secondary analysis of data derived from a previously published clinical trial investigating the effects of cognitive-behavioral therapy with exposure to real and virtual stimuli in individuals with specific phobia [10], which provides detailed information regarding participant recruitment, eligibility criteria, intervention procedures, stimulus presentation, study design, and primary clinical and neuroimaging outcomes. The current analysis specifically focused on pre-treatment functional connectivity patterns associated with variability in treatment response.

131 adults reporting specific phobia to small animals, were recruited through a public advertisement from the University of La Laguna (Tenerife, Spain). They provided written informed consent before starting the study, in accordance with ethical guidelines. This study was approved by the Ethics Committee for Animal Research and Welfare of the University of La Laguna (ref: CEIBA2012-0033)^1^.

Eligible participants (see Sect.  2.1. Participants) were randomised and given a code. Participants completed the assessment instruments (see Sect.  2.2. Instruments) and underwent an fMRI session with exposure to real or virtual 3D videos of the phobic stimuli. Following the initial assessment session, participants underwent CBT.

The CBT comprised eight weekly individual appointments, each lasting one hour.

- Session 1 introduced the therapist, outlined the programme, and began psychoeducation on specific phobias.
- Session 2 taught participants how to rate their anxiety with Subjective Units of Anxiety (SUA) and practised physiological down-regulation through controlled breathing.
- Session 3 reinforced breathing techniques, continued SUA monitoring, and introduced cognitive restructuring.
- Sessions 4–8 reviewed previous material and incorporated graded exposure to phobic stimuli, delivered as real or virtual images via 3-D goggles, while applying anxiety-regulation strategies to challenge distorted cognitions.

During these exposure sessions, participants engaged with the feared stimuli for approximately 27–32 min.

Fourteen clinical psychologists received specialised instruction on how to deliver the psycho-educational protocol and administer study measures. To maintain treatment fidelity, they attended supervision seminars every two weeks, led by senior experts who monitored and guided individual therapy sessions. Each clinician was randomly assigned, in a blinded fashion, to work with no more than five participants.

After CBT was completed, the assessment instruments were re-administered.

### Data analysis

#### Clinical response classification

First, the Reliable Change Index (RCI) was applied to assess whether each patient’s symptom reduction represented a statistically reliable improvement beyond what could be attributed to measurement error or natural variability [28].

The use of the Reliable Change Index (RCI) allowed classification of treatment response based on statistically reliable clinical change rather than arbitrary score differences. This approach takes into account both measurement error and test reliability, making it particularly suitable for identifying clinically meaningful improvement in pre-post treatment designs.

The RCI was computed by subtracting the S-R pre-test score from the S-R post-test score and dividing the result by the standard error of the difference between the two scores. An RCI greater than 1.96 indicates that the observed change is statistically reliable (*p* < 0.05). The RCI indicated reliable clinical improvement for all but two participants, whose change scores did not exceed the threshold of 1.96. Therefore, 29 participants remained.

**Fig. 1:**
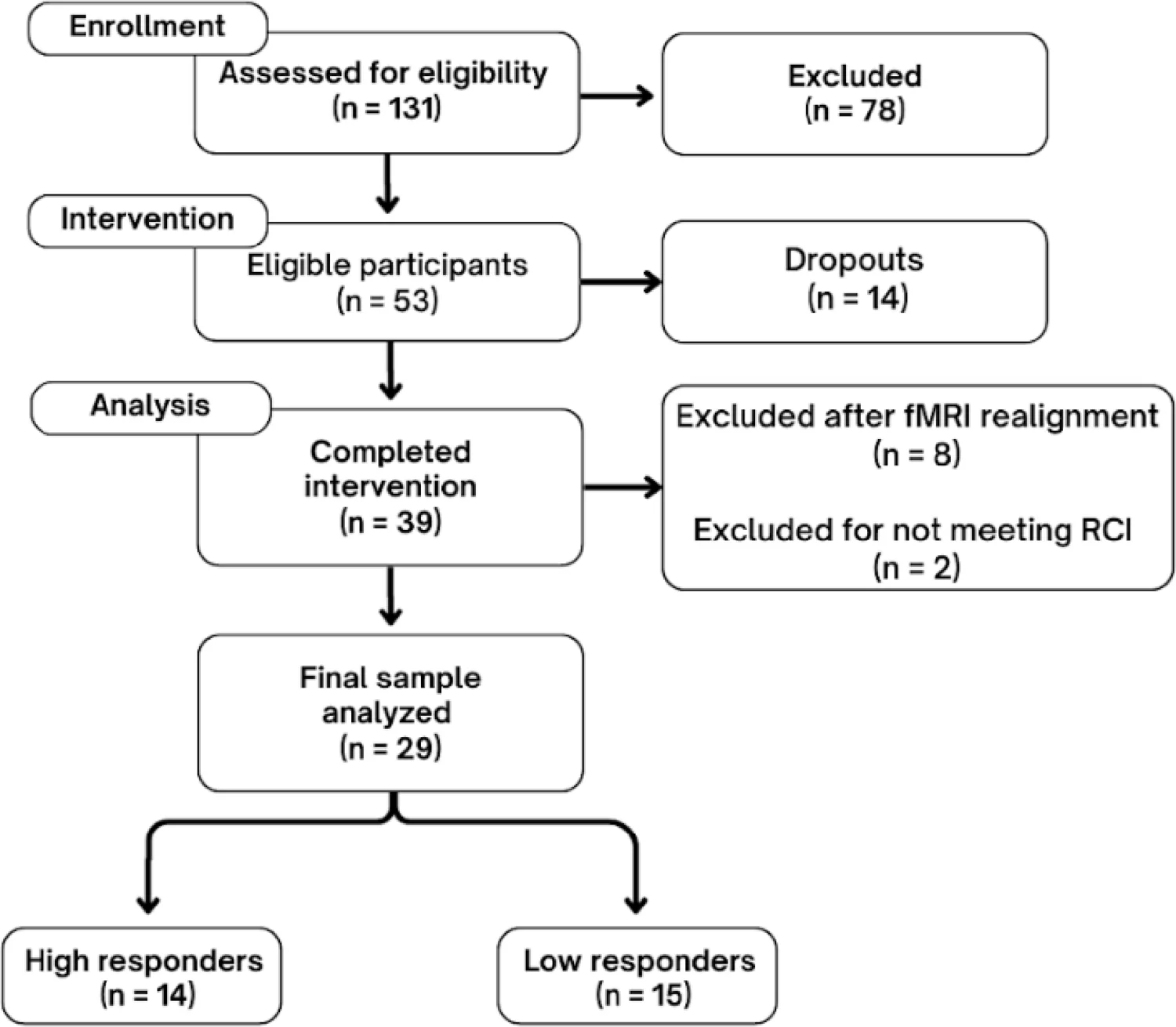
Flow chart

Once reliable improvers were identified, a second criterion was applied to classify the magnitude of clinical change. The post-treatment S-R score was compared against a cutoff defined as the stricter end of the normative mean, which is 30 (Endler et al., 1962) minus two standard deviations (2 × 7.5 = 15), yielding a cutoff value of 15. This cutoff was selected as a conservative distribution-based criterion intended to identify participants with clearly reduced post-treatment symptom levels rather than representing a universally standardized threshold for treatment-response classification. Post-treatment scores at or below 15 were classified as high responders, whereas scores above this cutoff were classified as low responders.

Following the second criterion the patients were allocated into two groups as follows:

- High responders = 14.
- Low responders = 15.

Using these criteria ensures that the strictest standards are met, resulting in highly reliable groups.

To further evaluate the performance of the classification procedure, agreement between RCI-based classifications and post-treatment S-R outcomes was examined. The RCI approach demonstrated high classification performance, yielding a sensitivity of 0.933, a specificity of 0.910, and an area under the receiver operating characteristic curve (AUC) of 0.910.

#### ROI selection and connectivity analysis

To investigate differences in brain functional connectivity between high and low responders prior to receiving CBT, we conducted a region-of-interest-to-region-of-interest (ROI-to-ROI) analysis using data from the first fMRI session (pre-treatment). ROI selection combined predefined large-scale functional networks and specific anatomically defined regions previously implicated in anxiety disorders, emotional regulation, and specific phobia in prior fMRI studies. Large-scale networks were included to examine distributed functional interactions potentially associated with CBT response, whereas specific ROIs were selected based on previous neuroimaging findings involving threat processing, cognitive control, reward-related processing, and fear extinction mechanisms. This combined approach allowed the exploration of both network-level and region-specific connectivity differences between high and low responders.

The ROI-to-ROI approach quantifies the correlation in BOLD signal time series between predefined brain regions, allowing identification of functional connectivity patterns associated with the clinical response groups.

#### Statistical analysis

The resulting pairwise ROI connectivity values were compared between groups (high vs. low responders) using two-sample t-tests. Statistical significance was assessed using a false discovery rate (FDR) correction for multiple comparisons at *p* < 0.05. Effect sizes were reported as Cohen’s d.

The analysis was performed for three sets of outputs:

- Simple effect of high responders (connectivity patterns within the high-response group during phobic stimulus exposure).
- Simple effect of low responders (connectivity patterns within the low-response group during phobic stimulus exposure).
- Direct group comparison (t-test: high responders vs. low responders).

## Results

fMRI data from 29 participants (14 high responders and 15 low responders) were analyzed. No significant differences were observed between high and low responders in baseline demographic or clinical variables, including age, sex, and baseline symptom severity measures.

**Table 1:** Baseline demographic and clinical characteristics of high- and low-responders groups

Participants (*n*)	Low responders	High responders
	15	14
Mean age (years)	35.07	35.00
Gender (women)	73.3%	85.7%
Baseline S-R score	37.53	41.21
Post S-R score	21.24	12.07
RCI	-7.11	-12.72

Independent t-tests between both groups were conducted to evaluate differences in connectivity between regions of interest (ROIs) from a whole-brain parcellation. Only differences that remained statistically significant after FDR correction (*p* < 0.05) were reported, all of them showing greater connectivity in low responders.

The analysis identified multiple connections in which the low-responder group exhibited significantly greater connectivity after false discovery rate (FDR) correction (*p* < 0.05), than the high-responder group. These included bidirectional associations between the supplementary motor area and the medial prefrontal cortex (MPFC) within the default mode network, as well as between the MPFC and regions within the sensorimotor network (superior sensorimotor area, bilateral precentral gyri) and the superior parietal lobule. Additionally, marked differences were observed between the nucleus accumbens (bilaterally) and regions within the language network, particularly the inferior frontal gyrus (triangular subdivision and language-related regions) and the superior temporal gyrus. The strongest effect (Cohen’s d ≈ 3.616) was found for the connection between the left nucleus accumbens and the left triangular inferior frontal gyrus. All reported effects were large in magnitude (Cohen’s d > 1.3), indicating robust group differences in connectivity patterns during exposure to phobic stimuli prior to treatment.

Table 2  summarizes the ROI-to-ROI connections showing significantly greater functional connectivity in the low-responder group compared to the high-responder group during exposure to phobic stimuli at baseline (pre-treatment).

**Table 2:** ROI-to-ROI

ROI Source	ROI Target	df = 27	*p*-FDR	d-Cohen
Supplementary Motor Cortex- Right	Default Mode Network (MPFC)	4	0.0225	1.649904
Accumbens Left	Network Language (IFG)	4	0.0052	1.824556
Accumbens Left	Inferior Frontal Gyrus, pars triangularis Left	9	0.0052	3.615668
Default Mode Network (MPFC)	Supplementary Motor Cortex- Right	4	0.0125	1.668484
Default Mode Network (MPFC)	Network Sensorimotor Superior	4	0.0125	1.63504
Default Mode Network (MPFC)	Precentral Gyrus Left	3	0.0434	1.367488
Default Mode Network (MPFC)	Precentral Gyrus Right	3	0.0434	1.363772
Default Mode Network (MPFC)	Superior Parietal Lobule Right	3	0.0491	1.311748
Network SensoriMotor Superior	Default Mode Network (MPFC)	4	0.0251	1.63504
Accumbens Right	Network Language IFG	4	0.0202	1.631324
Accumbens Right	Superior Temporal Gyrus Left	4	0.0202	1.519844
Accumbens Right	Inferior Frontal Gyrus, pars triangularis Left	4	0.0202	1.512412
Network Language IFG	Accumbens Left	4	0.0062	1.824556
Network Language IFG	Accumbens Right	4	0.0127	1.631324
Network Language IFG	Caudate Right	3	0.0466	1.3935
Inferior Frontal Gyrus, pars triangularis Left	Accumbens Left	4	0.0103	1.757668
Inferior Frontal Gyrus, pars triangularis Left	Accumbens Right	4	0.0217	1.512412
Inferior Frontal Gyrus, pars triangularis Left	Caudate Right	4	0.0217	1.501264

**Fig. 2:**
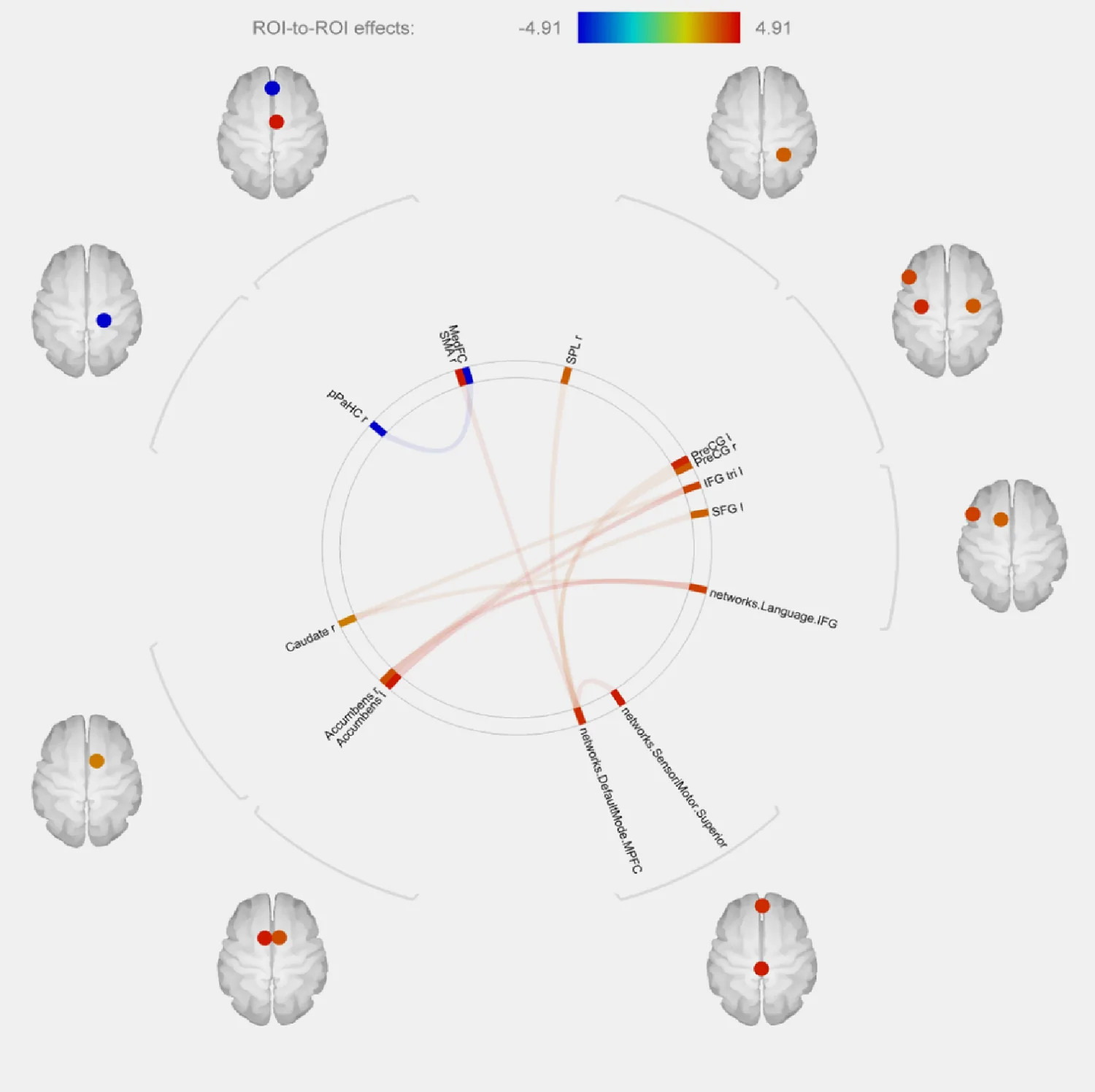
ROI-to-ROI effects

Additionally, the low responder group found greater overall bidirectional activity between the Parahippocampal Gyrus, anterior division Right and the Frontal Medial Cortex (df = 27, p-FDR = 0,0093, d-Cohen = 1.772532). In Fig. 2, the ROI to ROI effects are displayed

**Table 3:** Used in Fig. 2

MPFC/MedFC	Full Name
	Medial Prefrontal Cortex
IFG	Inferior frontal gyrus
STG	Superior temporal gyrus
PreCG	Precentral gyrus
SPL	Superior parietal lobule
pPaHC	Posterior parahippocampal gyrus
SMA	Supplementary motor área
Caudate	Caudate nucleus
Accumbens	Accumbens nucleus

## Discussion

The present study examined pre-treatment functional connectivity patterns in patients with specific phobia to small animals comparing those who later showed a high response to CBT and those with a lower response. Stringent criteria used for the creation of the groups allowed for the formation of homogeneous and statistically robust high- and low-responder groups, thereby maximizing the reliability of the neurofunctional comparisons performed (Table 2).

Significant group differences emerged, with low responders showing increased functional connectivity within language, cognitive-control, reward-related, and sensorimotor networks. Because fMRI data were collected prior to the intervention, these findings suggest that the observed connectivity differences are not a consequence of therapy but rather represent baseline neural characteristics that may be associated with differential treatment responses (Table 3).

Although responder groups were defined according to post-treatment outcome, connectivity analyses were based exclusively on pre-treatment data. Moreover, no significant baseline demographic or clinical differences were observed between groups, suggesting that the reported connectivity patterns are unlikely to be explained solely by pre-existing symptom severity differences.

Low responders showed greater connectivity involving language-related and motor/sensorimotor networks, suggesting altered interactions between reward-related, self-referential, cognitive-control, and motor-preparatory systems prior to treatment. These findings are broadly consistent with previous neuroimaging models implicating distributed functional networks in anxiety disorders and treatment response variability [29].

### Differential functional connectivity between groups

#### Language network connectivity

Hyperconnectivity was observed between the bilateral nucleus accumbens and nodes of the language network, including the inferior frontal gyrus (pars triangularis) and superior temporal gyrus, as well as the caudate nucleus. These findings suggest stronger integration between reward/motivation circuits and regions involved in verbal processing and cognitive control in low responders. This pattern could be related to increased engagement of verbal-conceptual and cognitive-control processes during threat-related stimulus processing. Similar fronto-striatal and prefrontal alterations have previously been associated with anxiety-related processing and treatment response variability in anxiety disorders [29].

Low responders also showed stronger connectivity between the left inferior frontal gyrus (pars triangularis) and the right caudate nucleus, as well as between the broader language network and the right caudate. The caudate is part of the dorsal striatum and plays a key role in habit formation, reinforcement learning, and action selection [30]. Increased fronto-striatal coupling may suggest greater reliance on over-controlled or less flexible cognitive and behavioral response patterns during phobic stimulus processing, although alternative interpretations related to salience attribution or attentional allocation should also be considered. While such heightened control might initially reduce threat exposure, it could also limit the flexibility required for fear extinction during CBT, potentially contributing to persistent avoidance-related processing and potentially interfering with processes involved in fear extinction and safety learning during exposure-based interventions [31–32]. This interpretation is compatible with previous models emphasizing the role of prefrontal and medial frontal regions in extinction-related processes and treatment response in anxiety disorders [29, 31, 32].

Such internal processing could maintain or even amplify negative appraisals, reinforcing avoidance-related cognitions and thereby limiting the corrective learning processes that are central to exposure therapy. Excessive coupling involving prefrontal and language regions could bias motivational systems toward avoidance responses, making it more difficult for patients to fully engage with feared stimuli during exposure tasks. Moreover, the inferior frontal gyrus has been implicated in inhibitory control [33–34], suggesting that hyperconnectivity may reflect an over-recruitment of regulatory mechanisms that paradoxically interfere with fear extinction by sustaining threat representations rather than allowing their responses to decrease naturally. Together, these findings may reflect altered integration among motivational, cognitive, and verbal networks that could be associated with reduced treatment responsiveness.

#### Motor areas connectivity

Low responders also exhibited increased connectivity between the medial prefrontal cortex (MPFC), a core node of the default mode network (DMN) [35], and several motor and sensorimotor regions, including the right supplementary motor area, bilateral precentral gyri, superior sensorimotor cortex, and the right superior parietal lobule. The MPFC is known to mediate self-referential processing, internal mentation, and valuation [36–37], whereas the sensorimotor and parietal regions are primarily involved in action planning, motor execution, and attentional control. Greater coupling between these regions may indicate that, when confronted with phobic stimuli, low responders engage in heightened self-focused and body-focused processing, accompanied by increased motor preparation and attentional allocation to the threat. This pattern may be compatible with increased anticipatory processing, heightened attentional allocation to threat, and greater motor readiness during phobic stimulus exposure, that may interfere with fear extinction learning during exposure-based CBT [29].

The involvement of motor and parietal regions suggests that low responders may allocate greater resources to action readiness and vigilance rather than to emotion regulation or stimulus reappraisal. This over-preparation could reduce the opportunity for corrective safety learning, since exposure therapy relies on sustained contact with the feared stimulus under conditions of reduced avoidance. Taken together, the observed connectivity pattern could be associated with less flexible threat-related processing and increased anticipatory engagement of motor-preparatory systems.

### Clinical and conceptual implications

The present findings suggest that low responders may present distinct pre-treatment functional connectivity patterns involving reward-related, language-related, and motor/sensorimotor networks compared with high responders.

The results therefore point toward a maladaptive pre-treatment connectivity state in low responders, marked by over-recruitment of self-referential and control networks that that could be associated with reduced flexibility in emotional processing during CBT.

Clinically, these results suggest that connectivity patterns may eventually contribute to understanding which patients show poorer treatment response and inform future research on individualized intervention approaches (e.g., mindfulness or pharmacological enhancers of extinction learning) to optimize CBT. Overall, the study emphasizes the importance of considering distributed functional networks to inform individual treatment approaches for specific phobia.

Although these interpretations are consistent with current neurocircuitry models of anxiety disorders and fear regulation [29, FULLANA], the present findings remain correlational and exploratory in nature. Therefore, the observed connectivity differences should not be interpreted as direct evidence of specific psychological mechanisms. Alternative explanations involving attentional processing, salience attribution, or generalized threat sensitivity should also be considered. Replication in larger and independent samples will be necessary to clarify the functional significance of these connectivity patterns.

### Limitations

Treatment response was measured primarily via the S-R Inventory, which captures symptom reduction but not broader outcomes such as real-world avoidance behavior or quality of life.

The sample included only right-handed adults with specific phobia to small animals, limiting generalizability to other phobia types or populations.

Although the final sample size was relatively small (*N* = 29), the observed Cohen’s d values were substantial, which mitigates concerns that the results may be attributable to statistical noise. Although the observed effect sizes were substantial, the relatively small sample size may limit the generalizability and stability of the findings. Small neuroimaging samples are more susceptible to effect-size inflation and reduced reproducibility, and therefore the present results should be considered preliminary until replicated in larger and independent cohorts.

Eye-tracking measures were not included during fMRI acquisition; therefore, subtle visual avoidance strategies during stimulus exposure cannot be completely ruled out.

The study included both filmed and virtually rendered 3D stimuli during fMRI acquisition. Although previous work suggests comparable neural engagement across these formats, the present study did not formally assess potential connectivity differences between stimulus types. Therefore, modality-specific effects cannot be ruled out.

The study did not include long-term follow-up assessments, limiting conclusions regarding the stability of treatment response and the explanatory value of baseline connectivity patterns over time.

Given the exploratory nature of the study and the absence of predictive modelling or external validation procedures, the present findings should not be interpreted as definitive predictive biomarkers of CBT outcome.

Future studies should use multimodal measures and longitudinal designs to confirm and extend these findings.

## Data Availability

The data supporting the findings of this study are available from the corresponding author upon request.

## Footnotes

1 The full study protocol is available from the corresponding author upon reasonable request.
